# Upregulation of the NLRC4 inflammasome contributes to poor prognosis in glioma patients

**DOI:** 10.1038/s41598-019-44261-9

**Published:** 2019-05-27

**Authors:** Jaejoon Lim, Min Jun Kim, YoungJoon Park, Ju Won Ahn, So Jung Hwang, Jong-Seok Moon, Kyung Gi Cho, KyuBum Kwack

**Affiliations:** 1 Department of Neurosurgery, Bundang CHA Medical Center, CHA University, Yatap-dong 59, Seongnam, Republic of Korea; 2 Institute Department of Biomedical Science, College of Life Science, CHA University, Seongnam-si, Gyeonggi-do Republic of Korea; 3 Soonchunhyang Institute of Medi-bio Science (SIMS), Soonchunhyang University, Cheonan-si, Chungcheongnam-do Republic of Korea

**Keywords:** CNS cancer, Cancer microenvironment

## Abstract

Inflammation in tumor microenvironments is implicated in the pathogenesis of tumor development. In particular, inflammasomes, which modulate innate immune functions, are linked to tumor growth and anticancer responses. However, the role of the NLRC4 inflammasome in gliomas remains unclear. Here, we investigated whether the upregulation of the NLRC4 inflammasome is associated with the clinical prognosis of gliomas. We analyzed the protein expression and localization of NLRC4 in glioma tissues from 11 patients by immunohistochemistry. We examined the interaction between the expression of NLRC4 and clinical prognosis via a Kaplan-Meier survival analysis. The level of NLRC4 protein was increased in brain tissues, specifically, in astrocytes, from glioma patients. NLRC4 expression was associated with a poor prognosis in glioma patients, and the upregulation of NLRC4 in astrocytomas was associated with poor survival. Furthermore, hierarchical clustering of data from the Cancer Genome Atlas dataset showed that NLRC4 was highly expressed in gliomas relative to that in a normal healthy group. Our results suggest that the upregulation of the NLRC4 inflammasome contributes to a poor prognosis for gliomas and presents a potential therapeutic target and diagnostic marker.

## Introduction

Glioma represents the most prevalent primary tumor of the central nervous system, with high morbidity and mortality rates. The standard therapy for gliomas comprises tumor resection and subsequent radiotherapy and chemotherapy with temozolomide in the adjuvant setting^1,2^. However, the efficacy of this multimodal therapeutic strategy is limited as a result of the overall resistance to therapy and the high risk of recurrence^3^, for which there are limited therapies, including with nitrosoureas and bevacizumab, with minimal clinical efficacy^4^. Therefore, a novel approach is greatly needed to address the extremely poor therapeutic results for patients with gliomas.

Mounting evidence indicates that the inflammatory microenvironment plays an important role in tumor development^5,6^. However, innate immunity regulating the inflammatory reaction in tumor microenvironments remains poorly characterized. Inflammasomes are critical intracellular multiprotein complexes of innate inflammatory pathways that are responsible for the production of interleukin (IL)-1β and IL-18^7,8^. Although inflammasomes are important for central nervous system diseases such as Alzheimer’s disease, multiple sclerosis, and traumatic injury, the role of inflammasomes in brain tumors remains unclear^9–11^.

Inflammasomes are formed by the assembly of cytosolic sensors (AIM2-like receptors and nucleotide-binding domain and leucine-rich-repeat-containing receptors, also known as NOD-like receptors [NLRs]), an adaptor protein apoptosis-associated speck-like protein containing a caspase recruitment domain (ASC), and an effector caspase, procaspase-1^7^. Two distinct signals are required for the activation of inflammasomes. The first involves pathogen- or danger-associated molecular patterns, including bacteria and viruses or reactive oxygen species, ATP, and DNA^12,13^, which are recognized by pattern recognition receptors such as Toll-like receptor or NOD family members on microglia, astrocytes, and macrophages in the central nervous system and prime the expression of pro-IL-1β and pro-IL-18^14^. The second signal involves tissue damage, metabolic dysregulation, ATP, and hyaluronan, which induce the cleavage of pro-IL-1β and pro-IL-18 to mature forms^15,16^.

Recent data indicate that inflammasomes play important roles in carcinogenesis and tumor progression^17^. Many endogenous and exogenous signals induce chronic inflammation and inflammasome activation in cancer^18^. We have previously reported that cellular glycolysis pathway, which plays an important role in cancer energy metabolism, are associated with NLRP3 inflammasome activation in macrophages under pro-inflammatory conditions^19,20^. However, the role of inflammasomes in tumors remains disputed.

In the present study, we comprehensively analyzed the immunohistological expression and distribution of inflammasome-associated components in tissues from glioma patients. We also performed a genome-wide analysis to assess the expression of inflammasome-associated genes and their association with the overall survival of patients diagnosed with gliomas included in the Cancer Genome Atlas (TCGA) database. Our data suggest that NLRC4 may represent not only a potential therapeutic target for gliomas, but also a biomarker for diagnosis and prognosis in brain cancer.

## Results

### Expression of the NLRC4 inflammasome in glioma patients

**Figure 1:**
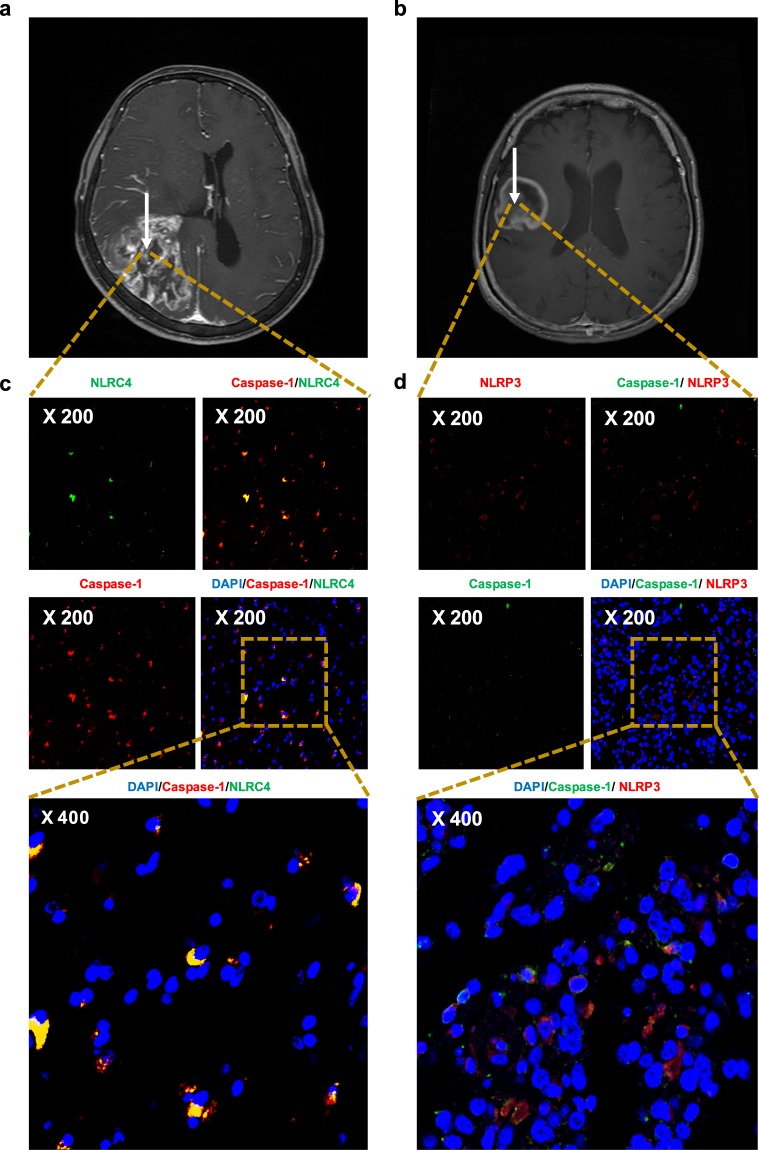
NLRC4 and NLRP3 inflammasomes are present and activated in brain tissue from glioma patients. Preoperative magnetic resonance imaging scans of 62-year-old (**a**) and 76-year-old (**b**) female patients with GBM. White arrows highlight tumor areas. (**c**) Human glioma specimens were analyzed by immunohistochemistry for the expression of NLRC4 (green) and caspase-1 (red). (**d**) Samples were stained for NLRP3 (red) and caspase-1 (green). Nuclei were stained with DAPI (blue).

Representative MRI results from patients with GBM are shown in Fig. 1a,b. Consistent with a previous report showing that the NLRP3 inflammasome is activated in patient-derived cell lines and microglia^17^, we found that the mRNA levels of *NLRC4* were significantly elevated in glioma patients (see Supplementary Fig. S1g). Thus, NLRC4 and NLRP3 were selected for immunohistochemical examination. Additionally, colocalization with caspase-1 was examined as a marker of inflammasome activation^18^. As shown in Fig. 1c,d (see also Supplementary Fig. S2), NLRC4 and NLRP3 were detected by immunostaining in the brains of glioma patients. Moreover, both proteins show colocalization with caspase-1.

Caspase-1 is present in the cytosol as an inactive zymogen^19^. In response to pathogen infection and tissue damage, pattern recognition receptors (PRRs) (NLRP3, NLRC4) oligomer is formed and it can recruit ASC. Then procaspase-1 can be recruited to the PRR-ASC complex by their CARD (caspase recruitment domain). This recruitment converts procaspase-1 into active caspase-1 via proximity-mediated cleavage^20^. Therefore, Fig. 1c,d (colocalization of NLR families and caspase-1) indicates that inflammasome were assembled and activated in the tumor tissues.

### High *NLRC4* expression is associated with poor overall survival in glioma patients

**Figure 2:**
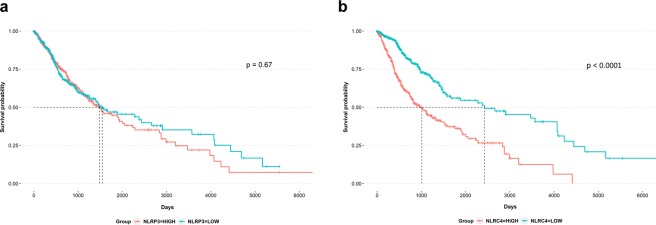
High *NLRC4* expression is associated with poor survival. Kaplan-Meier plots for comparing survival rates of glioma patients in relation to expression levels of *NLRP3* (**a**) and *NLRC4* (**b**) in the tumors based on the GBMLGG dataset from TCGA. Divided two groups by median value of each expression level.

To investigate NLRC4 and NLRP3 inflammasomes as potential prognostic markers in glioma patients, we compared *NLRP3* (Fig. 2a) or *NLRC4* (Fig. 2b) expression with survival using TCGA data. Kaplan-Meier survival plots indicated that glioma patients with high *NLRC4* expression had a significantly lower overall survival than those with low *NLRC4* expression, whereas the expression of *NLRP3* did not have a significant association with overall survival.

Sutterwala *et al*. reported that NLRC4 acts independently of inflammasome activation to suppress melanoma tumor progression^21^. However, the role of inflammasome differ among cancer types. Figure 2b showed that the median survival time of glioma patients with highly expression NLRC4 was significantly decreased compared to patients with low NLRC4 expression. In addition, we provided expression patterns of genes which are involved to NLRC4-mediated inflammasome including *IL1B* which is one of final product of NLRC4 inflammasome (Fig. S1). *IL1B* was significantly up-regulated according to poor prognosis group. On the other hand, previous report demonstrated that secreted IL-1β promotes chronic inflammation and induces glioma progression^17^. Collectively, these results indicate that NLRC4 inflammasome promotes glioma progression.

### High NLRC4 expression in astrocytes

**Figure 3:**
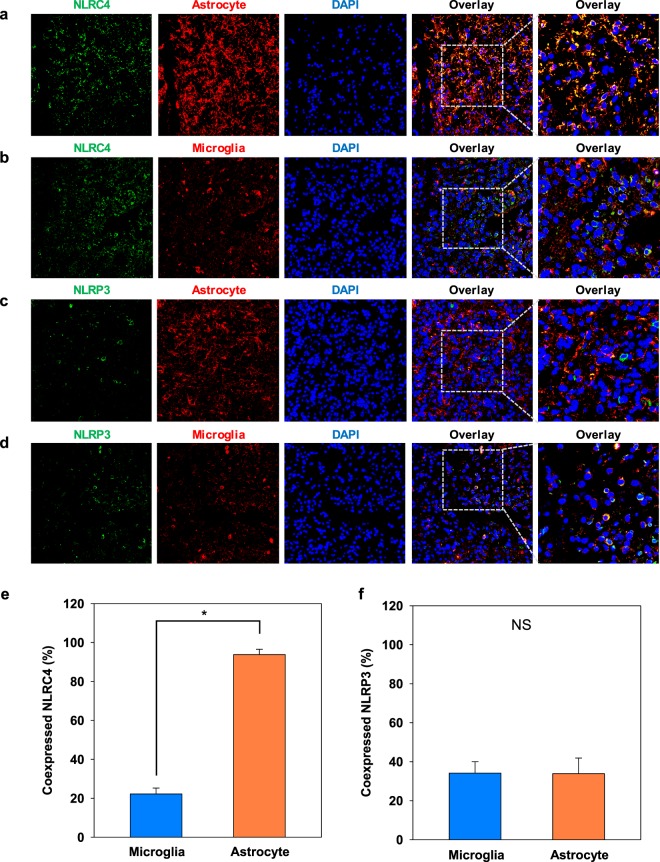
NLRC4 is highly expressed in astrocytes from glioma patients. Astrocytes (**a**,**c**) and microglia (**b**,**d**) were analyzed by immunohistochemistry for the expression of NLRC4 and NLRP3, respectively. Nuclei were stained with DAPI (blue). Co-expression of NLRC4 (**e**) or NLRP3 (**f**) with markers for microglia and astrocytes was quantified by ImageJ software. The values are means ± SEMs from three replicates (*n* = 8). *T-test p-value < 0.001.

Despite the study of NLRC4 and NLRP3 inflammasomes in several neurological diseases (Alzheimer’s disease, Parkinson’s disease, stroke, and multiple sclerosis), their roles in gliomas remain undefined. The results in Fig. 1 indicated that NLRC4 and NLRP3 inflammasomes are activated in glioma patients. To better understand inflammasome function in the glial compartment, we compared their expression among different cell types, mainly microglia (identified by staining for Iba1) and astrocytes (identified by GFAP expression). Confocal images showed that both microglia and astrocytes expressed NLRC4 and NLRP3 components (Fig. 3a–d), with strong overlap between NLRC4 and GFAP, as shown by the yellow color in the merged image. Quantification via analyses with ImageJ confirmed this colocalization of NLRC4 in astrocytes (t-test, p-value < 0.001), whereas NLRP3 was expressed similarly by both astrocytes and microglia (Fig. 3e,f).

### Expression of inflammasome-associated genes in glioma patients

As there is a report demonstrating that human malignant gliomas activate NLRP3 inflammasomes and express IL-1β^17^, we sought to further investigate the connection between inflammasomes and gliomas. Pairwise Pearson’s correlation matrix heat maps of the expression of genes in NOD like receptors signaling pathway (hsa04621) which is related to inflammasomes from the KEGG database are shown for normal solid tissue and LGG and GBM samples from the TCGA database (Supplementary Fig. S1a,c,e, respectively). We found that the genes necessary for the assembly of functional inflammasome complexes were positively correlated in glioma patients but not in normal individuals. Supplementary Fig. 2b,d,f show the nearest neighbors for components of the canonical inflammasome complex (*CASP1* [encoding caspase-1], *PYCARD* [encoding ASC], and *NLRP* [encoding NLRP]) with strong positive correlations. Genes encoding numerous NLR family proteins (*NLRC4*, *NOD1*, *NOD2*, *NLRP1*, *NLRP3*, *NLRP12*, and *NAIP*) and proinflammatory caspases/inflammasome adaptor molecules (*PYCARD*, *CASP1*, *CASP4*, and *CASP5*) were positively correlated with inflammasome complex genes in both LGG and GBM groups (Supplementary Fig. S1b,d,f) but not in normal brain (e.g., *NLRC4*, *NLRP3*, *PYCARD*, and *CASP1*; Supplementary Fig. S1b). Additionally, genes encoding proinflammatory cytokines regulated by the inflammasome (e.g., *IL1B*) and transcription factors (e.g., *NFKB1*) that prime inflammasome oligomerization and activation were significantly associated with inflammasome complex expression in LGG and GBM TCGA datasets. Furthermore, the expression levels of genes encoding NLR proteins (*NLRP12*, *NOD2*, *NOD1*, *NLRC4*, and *NAIP*), inflammasome adaptor molecules (*PYCARD*), and proinflammatory caspases (*CASP1*, *CASP4*, and *CASP5*) were significantly higher in glioma patients than in normal controls (Supplementary Fig. S1g). Moreover, the expression of inflammasome-associated genes was higher in GBM than in LGG. These results indicate that the inflammasome-associated pathway is activated in glioma patients.

### NLRC4 inflammasome in glioma patients

**Figure 4:**
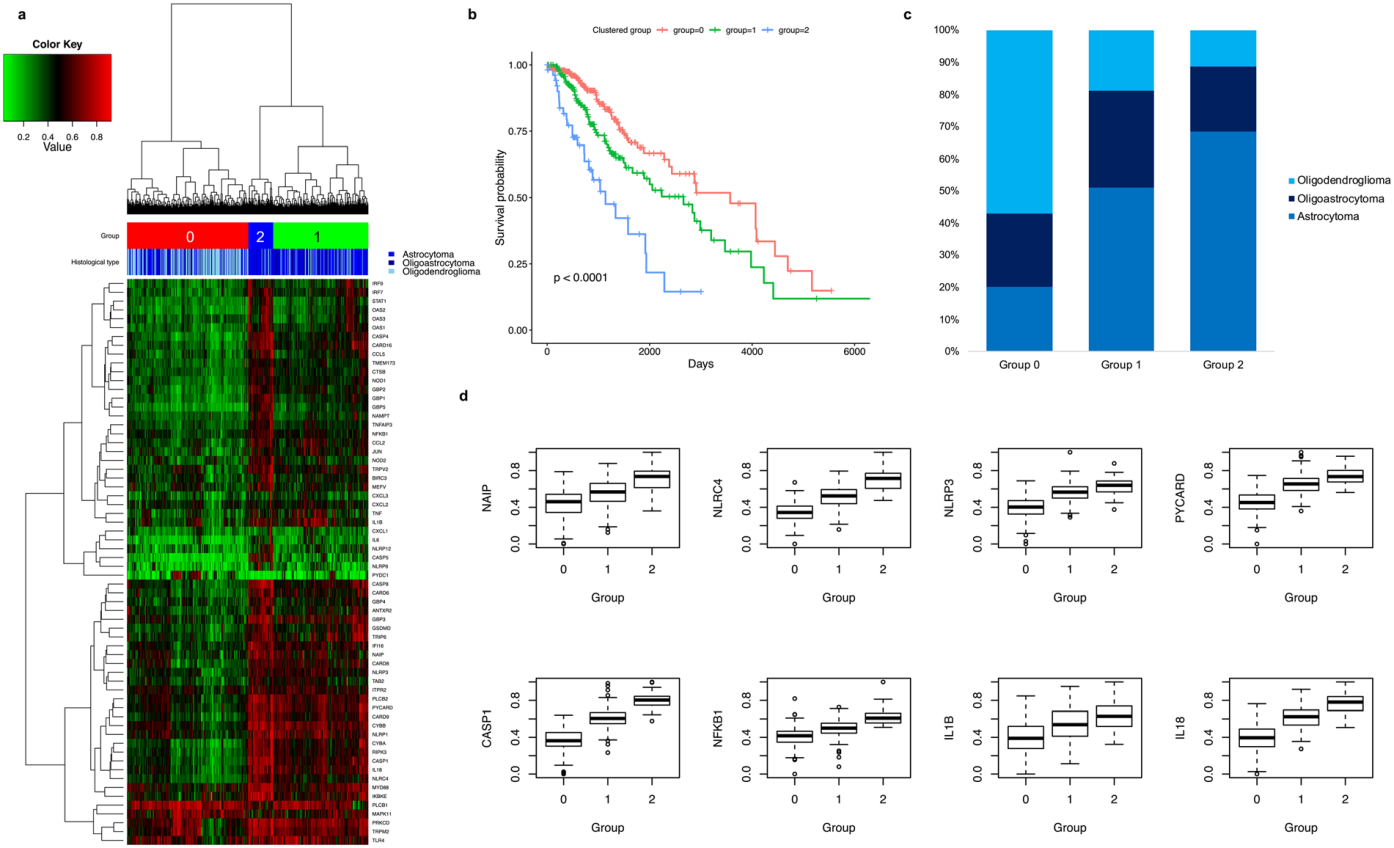
*NLRC4* is highly expressed in patients with astrocytomas and associated with poor prognosis. (**a**) Cluster map depicting the 64 mRNAs that were significantly differently expressed according to agglomerative clustering for inflammasome-associated genes from the TCGA LGG dataset (*n* = 525). Red represents high expression, green represents low expression, and the color scale depicted above the heat map displays histological types. (**b**) Kaplan-Meier survival curves for groups in panel A. (**c**) Plot of proportions of histological types by group. (**d**) Boxplots of expression levels with inflammasome-related key proteins including NLRC4 and NLRP3. The expression levels, log2[x + 1] RNA-seq expectation maximization (RSEM) counts, were transformed to minimum-maximum (0–1) normalized value.

To identify further potential associations between groups of inflammasome-associated genes with similar profiles and histological subtype and patient survival, we performed unsupervised hierarchical clustering of LGG data from the TCGA database (Fig. 4a). Most of the genes that were expressed at low levels clustered into group 0, whereas those with medium levels of expression were clustered into group 1 (Fig. 4a). A stronger association was found among highly expressed genes, such as *NLRC4*, as well as *NLRP3*, *PYCARD*, *CASP1*, *IL1B*, and *IL18*, which clustered in group 2 (Fig. 4d, Supplementary Tables S2 and S3). The expression levels of these genes (Fig. 4d) were higher in the astrocytoma subtype than in oligodendroglioma and oligoastrocytoma subtypes (Fig. 4c).

To investigate the prognosis of cancer with high expression of inflammasome-associated genes, overall survival of the patients was analyzed within the groups 0 to 2 (Fig. 4b). The best outcomes were associated with patients clustered in group 0, which exhibit low expression of these genes (Fig. 4d), whereas those with high levels of expression, in group 2, had the worst clinical outcomes (Pair-wise log-rank p-values < 0.01 after Benjamini-Hochberg corrections). These results indicate that high expression of inflammasome-related genes in glioma patients is associated with a poor prognosis (Fig. 4b,d and Tables S2 and S3). Especially, expression levels of the genes in NLRC4-mediated inflammasome pathway, including *NAIP*, *NLRC4*, *PYCARD*, *CASP1*, *NFKB1*, *IL1B*, and *IL18*, significantly increased according to the poor prognosis group (Fig. 4b,d).

**Figure 5:**
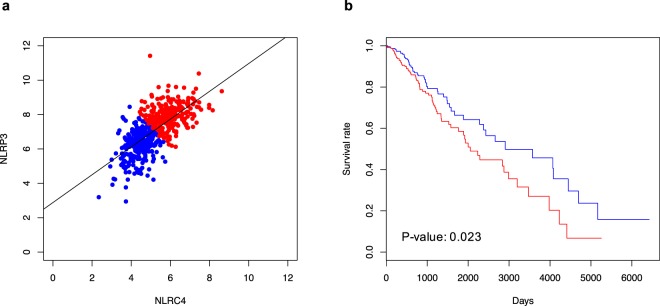
Correlated *NLRC4* and *NLRP3* expression in glioma patients (**a**) Dot plots from LGG dataset from TCGA for gliomas presenting correlations between *NLRC4* and *NLRP3*. (**b**) Kaplan-Meier survival plot for the TCGA glioma samples according to the co-expression levels of NLRC4 and NLRP3.

Although NLRC4 and NLRP3 typically form distinct inflammasome complexes^22–24^, recent reports demonstrate that the NLRC4 inflammasome recruits NLRP3 during *Salmonella* infection^25^, indicating that both are important for the activation of bacteria-mediated inflammasomes. To determine if this may also occur with inflammasomes in gliomas, we performed a correlation analysis using data from the TCGA dataset. *NLRC4* expression was positively correlated with *NLRP3* expression in GBM and LGG patients (correlation coefficient = 0.64, *p* < 0.001; Fig. 5a). In addition, a Kaplan-Meier survival analysis based on *NLRP3* and *NLRC4* expression demonstrated that patients with high expression of both had a significantly poorer prognosis (*p* < 0.023; Fig. 5b). Collectively, these data indicate that both NLRC4 and NLRP3 are upregulated in gliomas and suggest that they potentially associated with glioma progression.

## Discussion

There is emerging evidence that chronic inflammation in the tumor microenvironment impacts all stages of carcinogenesis and, depending on the proinflammatory cytokines, is associated with a poor prognosis^26^. Such inflammation, which involves chemokines, cytokines, and the extracellular matrix, is considered the seventh hallmark of cancer^5^. Inflammasomes contribute to the initiation of cancer-related inflammation and have various functions in tumorigenesis^27^. However, the roles of inflammasome components differ among cancer types^27,28^, inhibiting tumorigenesis in colitis-associated colon cancer^29,30^ and promoting the development of epithelial skin cancer and breast cancer tumor growth and metastasis^29,31^. Feng *et al*.^32^ showed that high NLRP3 expression was associated with poor survival in patients with oral squamous cell carcinoma, whereas the survival of patients with breast cancer was correlated positively with NLRP3 and negatively with NLRC4 as reported by Kolb *et al*.^28^. By contrast, NLRC4 expression was found to show no correlation with the survival of patients with hepatocellular carcinoma by Wang *et al*.^33^. Recently, Xiao-Feng *et al*. reported that NLRP3 inflammsome promotes glioma by regulating cellular proliferation, apoptosis and metastasis^34^. However, the clinical role of the inflammasome in gliomas is not known. The results of this study reveal that NLRP3 and NLRC4 inflammasomes are expressed and activated in gliomas and that high expression of NLRC4 in particular is associated with poor overall survival.

To better understand the role of inflammasomes in gliomas, their composition and activation in inflammatory cells should be characterized. Whereas microglia, the resident macrophages, account for only 10–15% of all cells in brain^35^, astrocytes, which maintain ion homeostasis and support neurons, are the most abundant^36^. Gustin *et al*.^37^ showed that NLRP3 inflammasomes are present and activated by lipopolysaccharide in microglia but not astrocytes in mouse brain. However, Freeman *et al*.^38^ showed that NLRP3 and NLRC4 inflammasomes are active in both microglia and astrocytes, with stronger NLRC4 expression in astrocytes, in a model of multiple sclerosis, similar to what we observed in samples from glioma patients in this study. Remarkably, the expression of NLRC4 was higher in the astrocytoma subtype than in oligodendroglioma and oligoastrocytoma subtypes (Fig. 4c,d) and was associated with poor survival (Fig. 4b).

**Figure 6:**
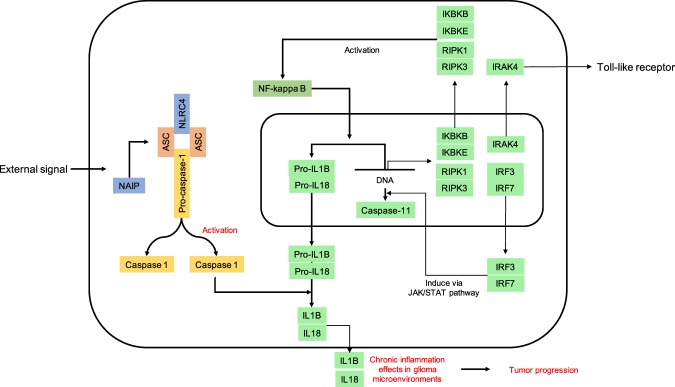
Schematic illustration of NLRC4 inflammasome pathway in glioma.

Although astrocytic NLRC4 has been implicated in Alzheimer’s disease and multiple sclerosis^38,39^, the present report using the TCGA glioma dataset is the first description of NLRC4 expression and its effects on the clinical prognosis in patients with astrocytomas (Fig. 4). We also provide the first characterization of inflammasome-associated transcripts in LGG and GBM patients. Overall, we observed positive correlations in glioma patients, but not normal controls, among genes encoding NLR family proteins, an adaptor molecule, and proinflammatory caspases, which are key components of the inflammasome (Fig. 6). Of note, patients with GBM showed high expression of many of these as well as of *IKBKB* and *IKBKE* (Supplementary Fig. S1g and Table S1), which encode kinases that relieve inhibition of NF-κB^40^. NF-κB regulates inflammasome activation by inducing the transcription of pro-IL-1β and NLRP3^41,42^. Recently, Irrera *et al*.^43^ blocked the NF-κB and NLRP3 inflammasome pathways with pharmacological inhibition of IKK-β (encoded by *IKBKB*), demonstrating its importance in inflammasome activation. Patients with GBM also showed high expression of *RIPK1* and *RIPK3*, which encode receptor-interacting serine/threonine protein kinases shown to drive NF-κB and inflammasome activation^44,45^, and *IRAK4*, which encodes IL-1 receptor-associated kinase 4, a protein kinase involved in innate immune response signaling from Toll-like receptors and associated with NLRP3 inflammasome activation^46,47^. Finally, our results show that tumor tissues in GBM patients highly express *IRF3* and *IRF7*, encoding interferon regulatory factors that can lead to the transcription of caspase-11, which is important for noncanonical NLRP3 inflammasome activation, via activation of the JAK/STAT pathway^48–50^.

In conclusion, the histological and bioinformatics analyses of tissue samples and TCGA data from glioma patients provide evidence that NLRC4 and NLRP3 inflammasomes are activated in gliomas. Moreover, high expression of NLRC4 was associated with poor patient survival. These findings contribute to the understanding of the role of the inflammasome in brain gliomas and suggest that NLRC4 is a potential therapeutic target and biomarker for diagnosis and prognosis in brain cancer.

## Materials and Methods

### Sample acquisition

Glioma specimens obtained during surgery from 11 patients (Table 1) at the Department of Neurosurgery, CHA Bundang Medical Center, CHA University (Republic of Korea), were stored at room temperature in neutral buffered saline. Among these patients, six had GBM (WHO grade IV), two had oligodendrogliomas (WHO grade II), one had a diffuse midline glioma (WHO grade IV), one had an anaplastic ganglioglioma (WHO grade III), and one had an anaplastic astrocytoma (WHO grade III). The median age of the patients was 51 years (range, 9–76 years).

**Table 1:** Glioma patients: clinical synopsis.

Patient no.	Gender/Age	Clinical diagnosis	IDH-1 mutant
1	F/76	Glioblastoma	Negative
2	F/51	Oligodendroglioma	Positive
3	M/28	Oligodendroglioma	Negative
4	M/67	Glioblastoma	Negative
5	F/9	Diffuse midline glioma (grade IV)	Negative
6	F/10	Anaplastic ganglioglioma (grade III)	Negative
7	F/59	Anaplastic astrocytoma	Negative
8	F/62	Glioblastoma	Negative
9	F/46	Glioblastoma	Negative
10	M/50	Glioblastoma	Negative
11	M/53	Glioblastoma	Negative

The Bundang CHA Hospital Institutional Review Board approval was obtained for this study (CHAMC 2016-04-012), and all patients signed informed consent forms. And all methods were performed in accordance with the relevant guidelines and regulations.

### Immunohistochemistry

Formalin-fixed paraffin-embedded glioma blocks were serially sectioned at a 4 µm thickness and deparaffinized in xylene. The sections were rehydrated after immersing in ethanol and incubated with goat serum to block nonspecific binding. The sections were then incubated overnight at 4 °C with primary rabbit monoclonal antibodies against NLRP3 and CARD12 or with mouse monoclonal antibodies against ionized calcium-binding adaptor protein 1 (Iba1) or glial fibrillary acidic protein (GFAP) (Abcam, Cambridge, MA). Secondary antibodies were applied for 1 h at room temperature. Images were acquired using a confocal laser scanning microscope (Zeiss LSM 880; Carl Zeiss, Jena, Germany).

### Description of TCGA data

Transcriptome sequencing and clinical data of GBM and low-grade glioma (LGG) were downloaded from the XenaTCGA database. The tumor statuses were LGG (*n* = 525) and GBMLGG (*n* = 702) dataset, and the sample types of GBMLGG dataset were recurrent tumor (*n* = 27), primary tumor (*n* = 670), and solid tissue normal (*n* = 5).

### Correlation analysis and expression patterns of inflammasome-associated genes in the KEGG pathway

The genes associated with the NLR signaling pathway (KEGG entry number hsa04621), involving two prototypic NLRs that induce caspase-1 via inflammasomes, were identified from the KEGG database. Pairwise Pearson’s correlation analyses between the expression of each of the NLR signaling pathway-related genes were performed and visualized as a cluster map based on a hierarchical method with Euclidean distances for recurrent, primary tumor, and solid tissue normal groups. To analyze gene expression patterns, agglomerative clustering (using ward method and Euclidean distance) was performed with whole NLR signaling pathway-related genes which are expressed over the 1 and divided 2 groups in LGG patients (n = 525) of TCGA. We extracted 64 differentially expressed genes between the 2 groups by t-test with Bonferroni corrections and absolute difference their expression levels over the 0.5. After extracting 64 genes, we performed agglomerative clustering again with the 64 genes using ward method and Euclidean distance after minimum 0 to maximum 1 normalized value.

### Statistical analysis

Pairwise log-rank tests with Benjamini-Hochberg corrections were performed to compare prognoses between the three groups which were divided by agglomerative clustering with 64 genes, and Kaplan-Meier plots were generated in all survival analyses. Two-dimensional K-means clustering of *NLRC4* and *NLRP3*, the two major inflammasome-related genes in the NOD-like receptors signaling pathway in KEGG, was performed, and the survival rates were compared between the two clusters.

## Supplementary information

Supplementary information

Supplementary Table S1

Supplementary Table S2

Supplementary Table S3
