# Supplementary material for: Upregulation of the NLRC4 inflammasome contributes to poor prognosis in glioma patients
Source: Sci Rep. 2019 May 27;9:7895. doi: 10.1038/s41598-019-44261-9 (PMC6536517; doi:10.1038/s41598-019-44261-9)
Supplement: Supplementary file 1 — Supplementary information [file 41598_2019_44261_MOESM1_ESM.pdf]

Upregulation of the NLRC4 inflammasome contributes to poor prognosis in glioma patients

Jaejoon Lim [1,#], Min Jun Kim [1,#], YoungJoon Park [2,#], Ju Won Ahn [2], So Jung Hwang [1], Jong-Seok Moon [3], Kyung Gi Cho [1,*], KyuBum Kwack [2,*]

[1] Department of Neurosurgery, Bundang CHA Medical Center, CHA University, Yatap-dong 59, Seongnam 463-712, Republic of Korea.

[2] Institute Department of Biomedical Science, College of Life Science, CHA University, Seongnam-si, Gyeonggi-do, Republic of Korea.

[3] Soonchunhyang Institute of Medi-bio Science (SIMS), Soonchunhyang University, Cheonan-si, Chungcheongnam-do, Republic of Korea.

***Correspondence to:**

KyuBum Kwack, Department of Biomedical Science, College of Life Science, CHA university, Seongnam-si, Gyeonggi-do, Republic of Korea. Tel: +82-31-881-7141, e-mail: kbkwack@cha.ac.kr or Kyung Gi Cho, Department of Neurosurgery, Bundang CHA Medical Center, CHA University, Yatap-dong 59, Seongnam 463-712, Republic of Korea. Tel: +82-31-780-5688, Fax: 82-31-780-5269, e-mail: sandori50@gmail.com

Supplementary figure legends

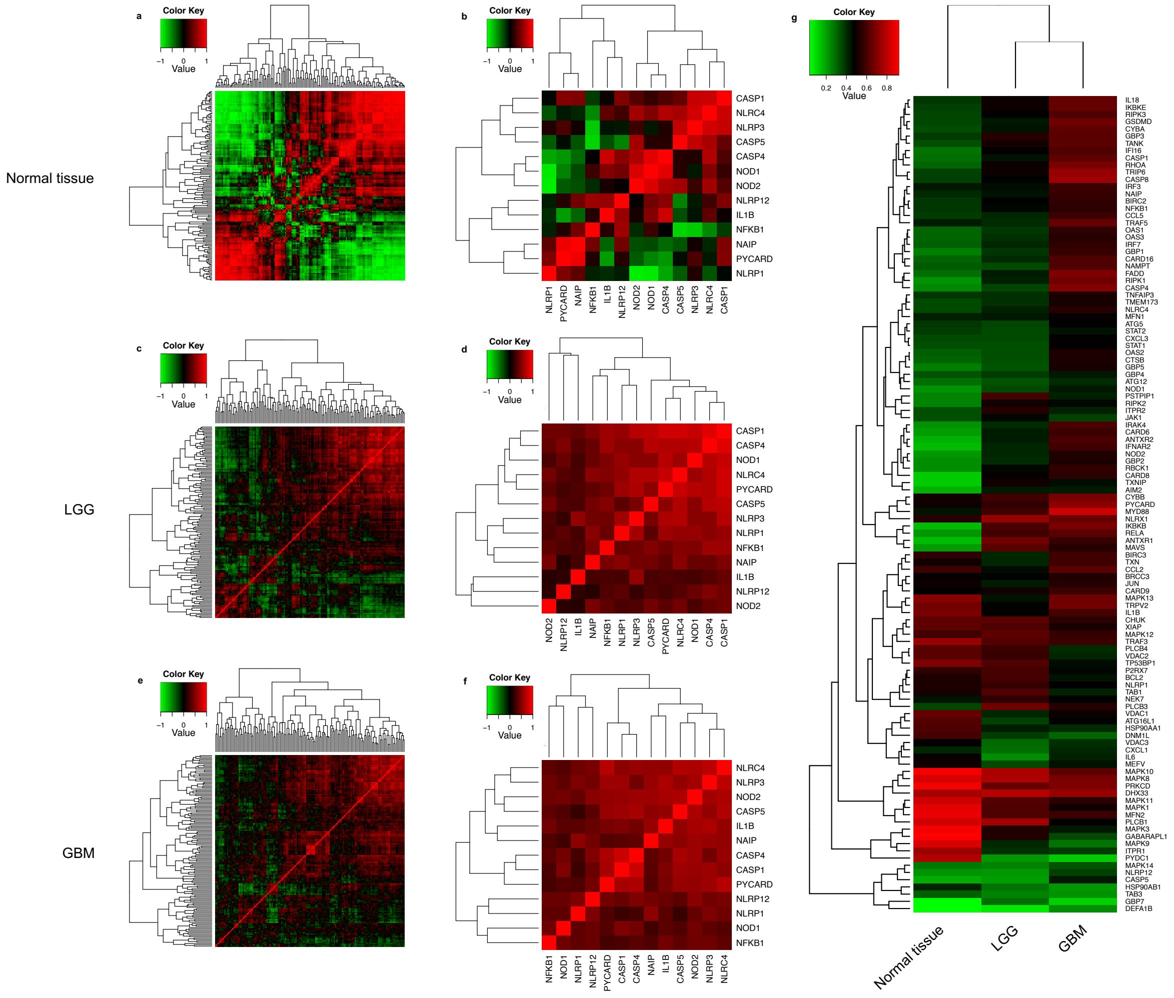

Supplementary Figure S1. Inflammasome-associated genes are well clustered, correlated, and overexpressed in glioma patients. The heat map and hierarchical clustering present the correlations between inflammasome-associated genes from brain samples from normal controls (a and b), LGG patients (c and d), and GBM patients (e and f) in the TCGA database. Genes are listed along the *x* and *y* axes. (g). Hierarchically clustered heat map analysis of relative gene expression of normal, LGG, and GBM patients in the TCGA database. Color from red to blue indicates high to low expression. The enlarged lists show the names of interested genes in the black dotted boxes, which indicate genes associated with the canonical inflammasome complex (a, b and c).

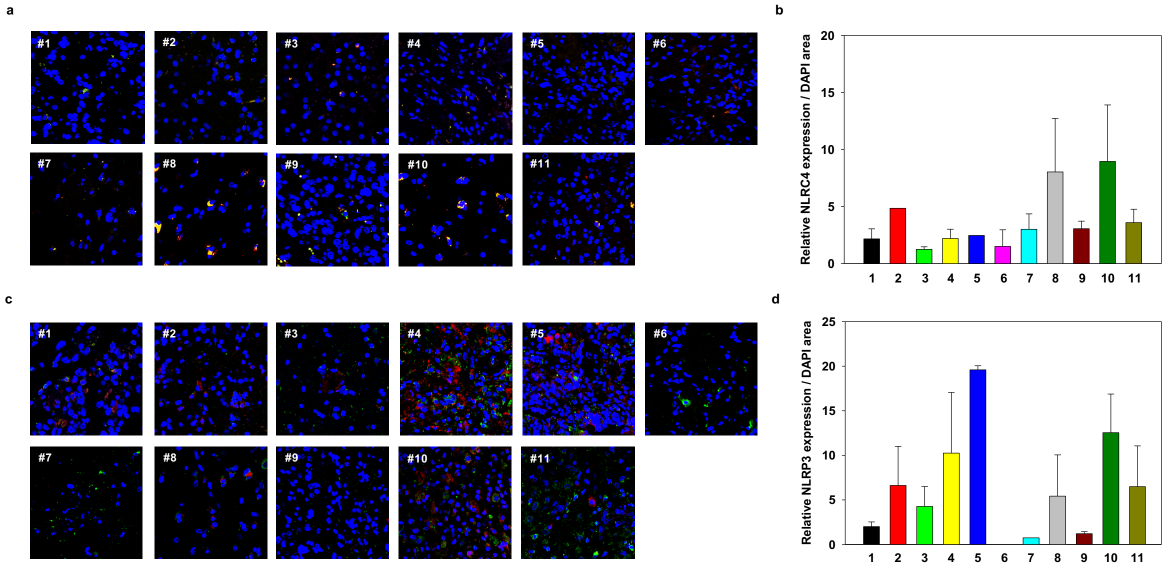

Supplementary Figure S2. Expression levels of NLRC4 and NLRP3 vary among patients. (a) Human glioma samples were analyzed by immunohistochemistry for the expression of NLRC4 (green) and caspase 1 (red). (c) Samples were stained for NLRP3 (red) and caspase 1 (green). Nuclei were stained with DAPI (blue). The relative expression of NLRC4 (b) or NLRP3 (d) is shown.

Supplementary Table S1. Average value of differential expressed genes between solid tissue normal, LGG, and GBM. ANOVA analysis was performed, and represented signficant genes after Bonferroni corrections.

Supplementary Table S2. Expression and clinical LGG data from TCGA (min 0 and max 1 normalized scale of log2-transformed of RPKM value). Group column indicates clustered by agglomerative clustering using ward and euclidean distance.

Supplementary Table S3. Average values and p-values of 64 genes. The 64 genes were extracted by ANOVA test for each group and adjusted with Bonferroni corrections for multiple comparisons.
